# An in Vitro Antimicrobial Activity of Ten Iranian-Made Toothpastes

**Published:** 2009

**Authors:** Mostafa Sadeghi, Shokrollah Assar

**Affiliations:** * Associate Professor, Department of Restorative Dentistry, School of Dentistry, Rafsanjan University of Medical Sciences, Rafsanjan, Iran; ** Instructor, Department of Microbiology, Immunology and Biology, School of Medicine, Rafsanjan University of Medical Sciences, Rafsanjan, Iran

**Keywords:** Anti-infective agents, Iran Toothpaste, Oral hygiene

## Abstract

**Background:**

Antimicrobial agents have been used as a chemotherapeutic agent to improve oral health. This in vitro study was carried out to determine the antimicrobial activity of ten Iranian-made toothpastes against commonly found bacteria in the oral cavity.

**Methods:**

The microorganisms used in this study were Streptococcus mutans, Streptococcus sanguis, Actinomyces viscosus and Candida albicans. Sterile discs impregnated with 10 Iranian-made toothpastes; Paveh, Saviz, Latifeh II, Bath, Darugar II, Darugar I, Close up, Tage, Pooneh III and Nasim, which were separately used on agar plates. Crest Cavity Protection toothpaste and Sterile pyrogenfree distilled water were used as positive and negative controls, respectively. The samples were tested in triplicate, at full strength, 1:1 and 1:3 dilutions. Inhibition zones were measured in millimeter after 48 hr. The data were analyzed by the ANOVA and t-test.

**Results:**

All tested toothpastes demonstrated an antimicrobial activity. The antimicrobial activity of Bath on S. mutans, Paveh on S. sanguis, Paveh, Saviz, Latifeh III and Darugar II on C. albicans were similar to the activity of Crest Cavity Protection. The antimicrobial activity of Pooneh III and Nasim on S. mutans, Bath on S. sanguis and A. viscosus, and Bath and Pooneh III on C. albicans were significantly higher and the others were significantly lower than the positive control. While, the activity of Crest Cavity Protection was the same as Pooneh III, it showed a weaker activity compared with Bath.

**Conclusion:**

Apart from Bath and Pooneh III, the other Iranian-made toothpastes tested in this study showed a lower antimicrobial activity compared to Crest Cavity Protection.

## Introduction

Tooth brushing with toothpaste is the most widely practiced form of oral hygiene in most countries.^1^ Twice daily brushing has significantly declined dental caries. Dental plaque is a biofilm on the tooth surface that plays an important role in the development of caries and periodontal diseases.^2^ While the mechanical removal of plaque on caries *per se* is equivocal, the maintenance of an effective plaque control program is the cornerstone of any attempt to prevent and control periodontal diseases.^3^ As a consequence, toothpastes provide an ideal vehicle for chemical adjuncts. A wide range of chemicals, mainly antimicrobial agents, have been added to toothpastes in order to produce a direct inhibitory effect on plaque formation.^1^,^4^ Clearly, most individuals find it difficult to maintain an effective level of plaque control and this is reflected in the levels of periodontal disease in the population. Approximately 50% of those aged 25 years and above had moderate periodontal diseases and there was a steady increase in the prevalence and severity of these diseases, and ultimately affect 15% of people aged 65 years and above.^3^ The addition of antimicrobial agents to toothpaste has been suggested as one possible method to improve the efficacy of mechanical tooth-cleaning procedures,^2^,^4^ aiding the control of dental plaque and preventing dental caries and periodontal diseases.^5^,^6^ When these substances are added to oral products, they kill microorganisms by disrupting their cell walls and inhibiting their enzymatic activity. They prevent bacterial aggregation, slow multiplication and release endotoxins.^6^–^8^ Several clinical studies have demonstrated the inhibitory effects of antimicrobial toothpastes on oral bacteria and gingiva.^1^,^4^,^9^ Also, the antimicrobial agents in toothpastes can significantly reduce contamination of toothbrushes.^10^,^11^ In a clinical study, two Iranian-made toothpastes, Pooneh and Nasim, were compared with Crest Regular. A significant reduction in plaque levels and gingivitis were observed in both groups, and there was no significant difference between them.^12^ The oral cavity is a highly contaminated area. There is approximately 10^8^ bacteria/ml of saliva. An oral hygiene product should be tested against microorganisms related to dental caries and periodontal diseases.^7^ At present, there is little information available on the antimicrobial potential of Iranian-made toothpastes. Based on these considerations, we conducted an in vitro study to assess the efficacy of antimicrobial properties of 10 Iranian-made toothpastes against bacteria commonly found in the oral cavity using agar diffusion method.

## Materials and Methods

In this experimental and single-blinded study, 10 different brands of toothpastes manufactured in Iran (three tubes of each brand) were purchased from different drug stores (Table 1). Crest Cavity Protection (Procter & Gamble UK, Weybridge, UK) served as positive, and sterile pyrogen-free distilled water (Darou Pakhsh Co, Tehran, Iran) used as negative control. Four oral microorganisms, streptococcus mutans (PTCC 1683), streptococcus sanguis (PTCC 1449), actinomyces viscosus (PTCC 1202) and candida albicans (PTCC 5027) were provided from Persian Type Culture Collection. The microorganisms were transferred into a tube containing Brain Heart Infusion Broth (BHIB) (Merck KGaA, Darmstadt, Germany). The incubation conditions were as follows: 37±0.5ºC, for 48 hr in candle jar for S. mutans, S. sanguis, and A. viscosus and at the same temperature for 24 hr for C. albicans. After incubation, sterile swabs were dipped into the bacterial suspension and inoculated on the following plates: S. mutans and S. sanguis were grown on Mueller Hinton blood agar (Merck KGaA), A. viscosus on BHI agar (Merck KGaA) and C. albicans on sabouraud dextrose agar (Merck KGaA). Antimicrobial activity of samples were determined by agar diffusion (Kirby-Baeur) method.^13^ We prepared three different amounts of each toothpaste at full strength (50 mg), 1:1 and 1:3 dilutions. Each toothpaste was transferred to a flask containing sterile distilled water and homogenized for 2 minutes using a vortex mixer and the above-mentioned dilutions were made. Sterile filter disks (Padtan Teb Co, Tehran, Iran) were impregnated with 50 mg toothpaste for full strength and some sterile filter discs soaked in diluted (1:1 and 1:3) samples. After drying discs in 60ºC oven for 2 hr, we placed them on the surface of inoculated agars. Afterwards, the plates were incubated at 37 ± 0.5ºC in an appropriate mentioned gaseous condition for 48 hours and then diameter of the inhibitory zones were measured in millimeters. The experiments were conducted in triplicate for three tubes of each toothpaste and the positive and negative controls. Twenty seven replicates were made for each microorganism per toothpaste. Then, considering 11 toothpastes, four microorganisms, and negative and positive groups, totally 1224 bacterial cultures were performed. Means and standard deviations of the diameters of inhibition zones were calculated. The data were analyzed using SPSS-14 software. One-way analysis of variance (ANOVA) was applied to determine the differences among three tubes of tested toothpastes and t-test was used for each toothpaste to compare with the controls at the significance level of P < 0.05.

**Table 1:** The tested toothpastes and their ingredients.

Toothpastes	Batch No	Manufacturer	Ingredients as Listed on Packages
Bath	1574	Iran Avandfar Co, Iran	Sorbitol, Carboxy methyl cellulose, Glycerin, Titanium dioxide, Sodium lauryl sulfate†, Sodium benzoate, Sodium monofluoro phosphate†, Hydrated silica, Tetrasodium pyrophosphate, Triclosan†, Deionizaed water, Flavour. Other Permissible additives.
Nasim	NA	Goltash Co, Isfahan, Iran	Sorbitol, Silica, Glycerin, Water, Sodium lauryl sulfate†, Carboxy methyl cellulose, monofluoro phosphate†, Flavour, Sodium fluoride†, Sodium saccharin, Sodium benzoate, Fragrance, Colour.-
Latifeh 2	152	Pakrokh Co, Tehran, Iran	Dicalcium phosphate, Carboxy methyl cellulose, Glycerin, Sorbitol, Sodium lauryl sulfate†, Essence, Sodium saccharin, Sodium monofluoro phosphate†, Silicon dioxide, Other Permissible additives.
Tage	180	Donyay ArayeshCo, Tehran, Iran	Sorbitol, Hydrated silica, Glycerin, Polyethylene glycol, Sodium lauryl sulfate†, Carboxy methyl cellulose, Sodiummonofluoro phosphate†, Titanium dioxide, Sodium saccharin, Sodium benzoate, Deionizaed water.
Darugar 2	NA	Kaf Co, Tehran, Iran	Dicalcium phosphate, Sodium-n-lauryl sarcosinate, Sodiumlauryl sulfate†, Sodium carboxy methyl cellulose, Methyl paraben, Bromo chlorophen†, Sodium monofluorophosphate†, Silica, Aerosil, Sorbitol, Glycerine, Propyleneglycol, Sodium saccharin, Flavour, Color, Deionizaed water.
Paveh	108	Toliddarou Co, Tehran, Iran	Silicon dioxide, Sorbitol, Glycerin, Sodium saccharin, Dicalcium phosphate dihydrate, Sodium carboxy methyl cellulose, Sodium lauryl sulfate†, Paraben, Essence, Deionizaed water.
Pooneh 3	NA	Goltash Co, Isfahan, Iran	Aqua, Silica, Sorbitol, Glycerin, Tetrasodium pyrophosphate, Sodium lauryl sulfate†, Carboxy methyl cellulose, Sodium monofluoro phosphate†, Essence, Titanium dioxide, Triclosan†, Methyl paraben, Sodium saccharin, Colour
Saviz	2	Sormah Co, Iran	Phosphate dicalcium dihydrate, Sodium monofluoro phosphate†, Sodium pyrophosphate, Glycerol, Sorbitol, Sweetener, Carboxy methyl cellulose, Flavour, Sodium laurylsulfate†, Aerosil 200, Preservatives, Water
Darugar 1	NA	Kaf Co, Tehran, Iran	Dicalcium phosphate, Sodium-n-lauryl sarcosinate, Sodiumlauryl sulfate†, Sodium carboxy methyl cellulose, Methyl paraben, Bromo chlorophen†, Methyl paraben, Sodium mono-fluorophosphate†, Aerosil, Sorbitol, Glycerine, Propylene glycol, Sodium saccharin, Flavour.
Close.up	NA	Unilever Iran Co, Iran	Sorbitol, Silica, Water, Polyethylene glycol, Sodium laurylsulfate†, Carboxy methyl cellulose, Sodium fluoride†, Sodium saccharin, Trisodium phosphate.
Crest Cavity Protection	NA	Procter & Gamble UK, Weybridge, UK	Aqua, Hydrated silica, Sodium lauryl sulfate†, Cellulose gum, Aroma, Carbomer, Glycerin, limonene, Sodium fluoride†, Sodium saccharin, Trisodium phosphate.

† Antimicrobial Agent NA=Non Available

## Results

The mean values and standard deviations of the microbial inhibition zones are shown in Table 2. The results indicated that all tested toothpastes and the positive control demonstrated a significant antimicrobial activity against the tested microorganisms as compared to negative control (P < 0.05), and the negative control showed no activi-ty. The antimicrobial activity of Bath on S. mutans, Paveh on S. sanguis, Paveh, Saviz, Latifeh III and Darugar II on C. albicans were similar to the activity of Crest Cavity Protection (P > 0.05). The antimicrobial activity of Pooneh III and Nasim on S. mutans, Bath on S. sanguis and A. viscosus, and Bath and Pooneh III on C. albicans were significantly higher and the others were significantly lower compared with the positive control (P < 0.05). While, the activity of Crest Cavity Protection was the same as Pooneh III, it showed a weaker activity compared to Bath. Other toothpastes indicated a lower activity than that of Crest Cavity Protection (P < 0.05). The data analyzed using ANOVA showed no significant difference among the three tubes of each brand regarding their antimicrobial activities.

**Table 2:** The mean ± SD diameter of inhibition zones (mm) in ten Iranian-made toothpastes against S. mutans, S. sanguis, A. viscosus and C. albicans

Toothpaste	S. mutans			S. sanguis			A. viscosus			C. albicans		
	100%^‡^	50%	25%	100%	50%	25%	100%	50%	25%	100%	50%	25%
Paveh	14.3±0.4	12.7±1.0	11.1±1.4	14.7±0.3	13.5±0.5	11.5±0.5	16.6±0.2	14.7±0.1	12.6±0.1	12.0±0.2	11.2±0.2	11.0±0.3
Saviz	10.7±0.3	9.6±0.3	8.8±0.8	13.7±0.3	12.8±0.3	10.3±0.3	13.5±0.2	11.8±0.1	10.1±0.4	12.3±0.3	11.6±0.3	9.9±0.2
Latifeh II	10.7±0.1	9.4±0.4	8.9±0.3	14.2±0.6	13.2±0.3	11.2±0.3	13.8±0.1	11.6±0.2	9.9±0.1	12.6±0.3	10.9±0.2	9.4±0.1
Bath	20.4±0.5	14.8±5.6	13.5±4.6	23.8±0.1	21.7±0.4	19.9±0.7	23.8±0.1	21.7±0.1	19.8±0.2	19.8±0.2	17.4±0.4	15.3±0.3
DarugarII	11.8±1.0	9.8±0.8	8.8±1.0	14.9±0.7	12.9±0.7	10.9±0.4	14.7±0.1	12.9±0.1	10.9±0.2	13±0.5	12±1	9.8±0.3
Darugar I	13.5±0.2	11.2±0.8	9.8±2.0	13.7±0.4	11.7±0.3	9.5±0.3	16.9±0.2	15.6±0.2	12.2±0.3	8.1±0.1	7.7±0.1	6.7±0.2
Close up	14.3±0.4	12.3±0.7	11.1±1.5	14.3±0.3	12.3±1.2	10.9±0.1	16.7±0.1	14.7±0.1	12.8±0.1	8.3±0.3	7.4±0.3	6.9±0.1
Tage	14.6±0.2	12.4±0.6	11.0±1.6	14.5±0.3	12.9±0.4	10.4±0.5	16.6±0.1	14.8±0.1	12.6±0.1	8.4±0.1	8.3±0.3	6.9±0.2
Pooneh III	11.0±0.2	9.3±0.5	8.1±0.7	22.3±0.6	20.6±1.0	18.3±0.5	13.6±0.1	11.6±0.2	9.9±0.1	21.0±0.1	17.6±0.5	15.2±0.2
Nasim	8.6±0.3	7.2±0.2	6.9±0.4	19.9±0.7	18.4±0.6	16.3±0.5	11.8±0.1	9.3±0.3	7.8±0.2	10.5±0.1	9.9±0.3	8.3±0.2
Positive Control	16.2±0.5	14.1±0.5	12.9±1.5	16.8±0.3	14.8±0.7	12.9±0.9	19.6±0.2	17.5±0.2	15.5±0.4	12.7±0.2	10.1±0.1	8.4±0.3
NegativeControl	00	00	00	00	00	00	00	00	00	00	00	00

## Discussion

S. mutans and S. sanguis are the most dominant bacterial species in dental plaque and the major pathogens of dental infection. Actinomyces spp. are commonly found in dental caries as secondary invaders, contributing to the progression of the lesion. Followed by primary invaders, oral cavities are vulnerable for secondary invaders like C. albicans and species of actinomyces.^7^,^14^,^15^ The viridans streptococci can enter the bloodstream through an oral infection wound or an extraction site, cause between 40 and 50 percent of cases of endocarditis.^16^ It is known that a balance exists in each person’s oral microbial population. If that balance is lost, opportunistic microorganisms can proliferate, enabling the initiation of disease processes.^14^ The use of a toothpaste as an adjunct to tooth brushing may assist oral hygiene practices in a number of ways. It may prevent plaque formation by interfering with bacterial adherence to the tooth surface and reducing salivary bacterial numbers.^3^,^4^,^8^ In the present study, three tubes of each toothpaste were purchased, cultured in three concentrations and triplicates, so the data obtained with extreme accuracy. Crest Cavity Protection toothpaste was used as positive control as it is approved by FDA. The antimicrobial agents in tested toothpastes include triclosan, bromochlorophene, sodium lauryl sulfate (SLS), sodium mono-fluorophosphate (MPF), and sodium fluoride (SF). Although SLS existing in these toothpastes is a detergent, it is also known to have antibacterial and plaque inhibitory activities^2^ (Table 1). The diffusion method can be used as a preliminary test for detecting antimicrobial activity in substances or products. Since the diffusion phenomenon depends on each substance’s physical-chemical properties, as for example its diffusion coefficient, as well as the medium where the diffusion occurs, it is possible to obtain a qualitative indication of antimicrobial activity.^7^

Therefore, the toothpastes that having the largest microbial inhibition zone and thus, probably the strongest antimicrobial properties may not be necessarily superior to those found to have smaller diameter inhibition zones.^14^ The results showed that antimicrobial property of Pooneh III was similar to and Bath higher than that of positive control toothpaste. This can be related to the presence of triclosan, sodium MPF and SLS in these products. The toothpastes containing more than one antimicrobial agent had higher activity against microorganisms. Triclosan as a major chemical ingredient possesses significant antibacterial activities. Triclosan is a chlorophenol derivative, kills germs by interfering with the enzymes required for fatty acid synthesis.^3^,^4^ A systematic review indicated that a toothpaste containing triclosan/copolymer provides a more effective level on plaque control and periodontal health than conventional fluoride toothpaste.^17^ Also, a clinical study according to O’Leary index showed that cleaning efficiency of Darugar was better than that of Crest, Paveh, and Pooneh. Cleaning efficacy of Nasim was the same as that of Crest while Golpasand and Saviz were weaker than Crest in this regard.^18^ Another clinical study showed that Pooneh and Nasim toothpastes were proved to be effective in plaque re-growth prevention; however, they concluded Crest Complete in their short-time study yielded more satisfactory results.^19^ The fluoride tooth paste reduces the number of streptococcal colony forming units of dental plaque^9^ despite the fact that fluoride was added to the toothpastes first with aiming to preserve the product and then to protect the teeth.^7^ Antimicrobial mechanisms of toothpastes containing fluoride (such as MPF and SF) are through interfering the glucose transport, carbohydrate storage, extracellular polysaccharide formation and acid formation by oral streptococci.^9^ Bromochlorophene is one of the antimicrobial agents, which is known to be an effective bactericide and a potential plaqueinhibitory agent. Clinical evidence showed that toothpastes containing bromochlorophene have an inhibitory effect on plaque accumulation and also reduce caries in both animals and humans.^20^ The main differences between the tested toothpastes and Crest Cavity Protection can be the type and concentration of antimicrobial agents. Most commonly used and recommended by the WHO, ADA and FDI are fluoride and triclosan. The regular evaluation of the efficacy of the fluoridated toothpaste by the private laboratory have been recommended by the WHO.^21^ There was optimum antimicrobial activity at full strength and linear reduce with decrease of dilution. The antimicrobial activity of toothpastes ingredients may deteriorate with time and such manufacturers look to ensure adequate shelf life for their products of at least six months.^2^

## Conclusion

Apart from Bath and Pooneh III, the other Iranian-made toothpastes tested in this study, showed a lower antimicrobial activity compared to Crest Cavity Protection. Based on our results, we strongly suggest toothpaste manufacturers take into consideration to improving the antimicrobial properties of their products.
